# The Book World of Medicine and Science

**Published:** 1898-07-09

**Authors:** 


### The Book World of Medicine and Science.

Allbutt's System of Med c ne. Volume V. (London: Macmillan and Co. 1898. Price 25s. net.)

Second Notice.

The second half of this volume is devoted to diseases of the circulatory system. Commencing with a most lucid description of the general features of the blood by Professor Michael Foster, there follow chapters on the clinical examination of the blood by Dr. Monckton Copeman and on cardiac physics by Dr. C. S. Sherrington. Every year something new comes to the front in regard to examination of the blood, and those who think that counting corpuscles is the beginning and end of it had better read Dr. Copeman's article. They will be considerably enlightened. Dr. Sherrington's article on cardiac physics is short but sufficient. One little sentence contains the keynote to so much that is of importance in cardiac pathology that it will bear quotation. "The main portion of the work of the heart is expended immediately, not on moving the blood through the vessels, but in stretching the arterial wall." Of course this is well understood by everyone who thinks on the subject, but it is far too often overlooked in discussing the work of the heart. Professor Allbutt contributes articles on chlorosis, on functional disorders, and on mechanical strain of the heart, and a very complete essay on diseases of the aortic area of the heart. Each of these articles serves well to illustrate not only the subject in hand but the author's literary method. There is no doubt that these articles are among the most readable in the volume. His semi-playful way of dealing with the most serious subjects, the aptness and yet the oddity of some of his illustrations, and the manner in which great truths are, so to say, taken for granted as between him and his readers, not only give piquancy to his writing, but render it highly suggestive.

Speaking of the murmurs of chlorosis, he describes the occurrence of true mitral regurgitation in these cases, and after describing the investigations of Professor MacAlister in regard to the large part taken by the auriculo-ventricular muscular structures in closing that orifice during systole he says, "On inspecting the model one began to wonder whether valves were not luxuries rather than necessaries; for the sphincter fibres, contracting during the ventricle, seemed to reduce the orifice almost to an imperceptible chink." In regard to treatment Professor Allbutt declines to admit that chlorosis is an obstinate disease if only it is properly treated, and goes on to maintain that the proper treatment is the apparently simple one of giving iron. This, however, is to be remembered, that "no careful prognosis can be given without repeated examinations of the blood," for "it is more than possible that many cases of chlorosis recorded as aberrant or peculiar were not cases of chlorosis at all." Failure to obtain success by the use of iron seems to be due to one of two causes, "first that iron failed of success because given in insufficient quantity; and, secondly, that the treatment was not continued long enough," and the latter is the most important. In regard to quantity, he says: "Iron must be given with a liberal hand; the quantity of the metal is more important than the particular preparation." Nevertheless, the quantity which Professor Allbutt gives does not appear to us to be very extraordinary. He prefers the sulphate, alone or with aloes, and he decries the addition of an alkali as being "quite useless." But three grains of sulphate of iron per day, running up to nine grains per day divided into three doses, does not seem anything out of the way—not half the quantity, in fact, that is recommended in Blaud's original prescription. The following remark is worth bearing in mind as explaining much inefficient drugging: "I have often suspected that incurable chlorosis may mean insoluble pills; pills made up, for instance, with gum tragacanth and the like become as hard as pebbles, and about as useful to the patient." He very rightly recommends the use of "jeloids," an admirable preparation of carbonate of iron in gelatine. In his article on functional disorders of the heart Professor Allbutt is peculiarly happy. To the purist, as he says, the vulgar distinction between functional and structural disease is a false one. Yet that functional disorder can exist short of what we can properly call disease is clear enough; nay, we may say that even between functional disorder and health no definite line can be drawn. "Is there a man so stoutly knit whose inhibitory nerves are so powerful and alert that, in passion or 'twixt doubtful fear and feeble hope,' he has never felt his mouth climb into his throat?" While maintaining the distinction in this sense between functional and organic cardiac disease, it is asked may not the one lead to the other? "Whether a purely functional disorder, by damnable iteration, can hammer disease, as it were, into a harassed organ is hard to say; as yet we can only say that in many cases a lifetime of functional disorder of no little persistency is not long enough to bring this event about, and perhaps that is the usual issue; on the other hand, it seems no less certain that perennial depressing causes, exile or bondage in an invisible Babylon, may induce degenerative changes in the heart and blood vessels" Professor Allbutt well says that cardiac neuroses are nearly always part of general neurosis, and his remarks on the evil effects of repeated dram-drinking in such cases are well worthy of attention. On this subject, however, he speaks the results of common sense and experience when he says, "Some cordial these patients will have, perhaps ought to have; they are frightened out of their wits, and a stimulant seems their only help." As a temporary help, therefore, various carminatives are advised. All through these articles we find a curiously vivacious and unconventional style, while behind it we can easily see of what a large experience it is the expression. The article by Dr. Sansom on diseases of the mitral valve is very good, but is more sober reading, while those on the various disturbances of the blood and the smaller blood vessels, the various forms of anæmia, hæmophilia, purpura, scurvy, infantile scurvy, hæmoglobinuria, leucocythæmia, and dropsy are of great excellence. The volume fully maintains the reputation gained by its predecessors.

